# Partial Reprogramming Exerts a Rejuvenating Effect on Human Mesenchymal Stem Cells That Underwent Replicative Senescence in Culture

**DOI:** 10.3390/ijms252312533

**Published:** 2024-11-22

**Authors:** Julia Ivanova, Mariia Shorokhova, Natalia Pugovkina, Irina Kozhukharova, Larisa Alekseenko, Nikita Guriev, Ivan Kuneev, Alisa Domnina, Tatiana Grinchuk, Victoria Zemelko, Olga Lyublinskaya

**Affiliations:** Department of Intracellular Signaling and Transport, Institute of Cytology, Russian Academy of Sciences, Tikhoretskii pr. 4, St. Petersburg 194064, Russia; shili-mariya@yandex.ru (M.S.); natalia.pugovkina@gmail.com (N.P.); kojuxarova@mail.ru (I.K.); al.l.l@mail.ru (L.A.); guriev.nik.and@gmail.com (N.G.); kynejev@gmail.com (I.K.); aldomnina@mail.ru (A.D.); grintat@bk.ru (T.G.); vzemelko@mail.ru (V.Z.); o.lyublinskaya@mail.ru (O.L.)

**Keywords:** partial reprogramming, mesenchymal stem cells, replicative senescence, rejuvenation, Sendai virus, Yamanaka factors

## Abstract

Mesenchymal stem/stromal cells (MSCs) are becoming increasingly important for biomedical applications, such as cell therapy, disease modeling, and drug screening. At the same time, long-term cultivation, which is necessary to prepare a sufficient amount of cellular material for therapeutic and research purposes, is accompanied by the development of replicative senescence. Partial reprogramming emerged as a novel method that shows promising results in the rejuvenation of cells in vitro and in vivo; however, it has not yet been applied for human MSCs that have undergone replicative senescence in culture. In the present study, we subjected senescent human endometrial MSCs to partial reprogramming using Sendai virus vectors containing genes encoding Yamanaka transcription factors Oct4, Sox2, Klf4, and c-Myc. Characterization of the MSCs 5 days after transduction showed the loss of key markers of senescence: the youthful morphology was restored, the expression of senescent-associated β-galactosidase and the number of double-strand DNA breaks decreased, proliferation was activated, and the DNA damage response was enhanced. Further, using an in vitro wound-healing assay, we demonstrated that conditioned medium from partially reprogrammed MSCs showed higher therapeutic activity than that from senescent cells. However, a biosafety test revealed the presence of viral components in conditioned medium, which caused the agglutination of erythrocytes. Collectively, our data suggest that partial reprogramming is a potentially effective strategy for the rejuvenation of cultured MSCs in late passages but requires the use of virus-free protocols, such as chemical reprogramming.

## 1. Introduction

Mesenchymal stem/stromal cells (MSCs) are multipotent clonogenic cells of mesodermal lineage. Over the past decade, MSCs have emerged as an effective tool for use in regenerative medicine and biomedical engineering due to the ease of their derivation and in vitro expansion, as well as their differentiation potential, immunomodulatory, and angiogenic paracrine activity [1,2]. At present, MSCs have shown encouraging results in clinical trials, but their application has some limitations, one of which is the need for the large amounts of cells required for transplantations [3]. MSCs can be propagated by culturing; however, repeated passaging of these cells leads to the development of a replicative senescence, which is defined as a state of permanent growth arrest after a certain number of cell divisions.

To date, there is a lot of evidence that cellular senescence broadly contributes to the development of organismal aging in several aspects, such as the promotion of tissue disfunction, the acquisition of a senescence-associated secretory phenotype (SASP), and stem cell pool exhaustion [4,5]. That is why the search for the causes of cellular senescence and possible approaches to prevent or reverse it is nowadays a focus of intense research [6]. One of the most promising methods is based on the technique of reprogramming of the adult cells into a pluripotent state. Initially, cellular senescence was considered a roadblock to this process [7]. Nevertheless, several groups soon showed that cells derived from old donors or that underwent senescence after long-term cultivation can also generate induced pluripotent stem cells (iPSCs), similar in their characteristics to iPSCs from young donors [8,9]. Subsequent differentiation of the obtained iPSCs resulted in the formation of progeny cells lacking age-associated markers, thus proving the possibility of cell rejuvenation by passing through the pluripotency stage. In 2016, Ocampo and colleagues proposed a new strategy of rejuvenation: cells and organs can be revitalized in vivo and ex vivo by partial reprogramming without the need for complete dedifferentiation to pluripotency [10]. In this groundbreaking study, it was demonstrated that cyclic partial reprogramming erases the cellular markers of senescence in mouse and human cells in vitro, as well as ameliorating the signs of aging and expands the life span of progeria mice. For reprogramming, a doxycycline-inducible cassette containing genes encoding the Yamanaka pluripotency transcription factors (Oct4, Sox2, Klf4, and c-Myc–OSKM) has been employed. Since then, a number of studies have been published both on mouse models in vivo and on human and murine cell lines ex vivo, confirming that partial reprogramming can be an effective rejuvenation strategy (the studies revised in [11]). Among others, there has been evidence of rejuvenation of MSCs from aged mice. However, there is limited knowledge about this issue in human MSCs that have undergone replicative senescence in culture [12]. Dr. Göbel and colleagues attempted to delay the onset of replicative senescence by expressing OSKM, LIN28, and shRNA for p53 in human MSCs at early passages. However, this approach did not result in the extension of cell expansion in culture or delay in the acquirement of the senescence-associated phenotype [13].

In this study, we report that the application of a partial reprogramming method can ameliorate senescence-associated markers and enhance the therapeutic activity of human MSCs that have undergone replicative senescence after prolonged culturing.

## 2. Results

### 2.1. Partial Reprogramming Leads to Erasure of Replicative Senescence Markers in MSCs

**Figure 1:**
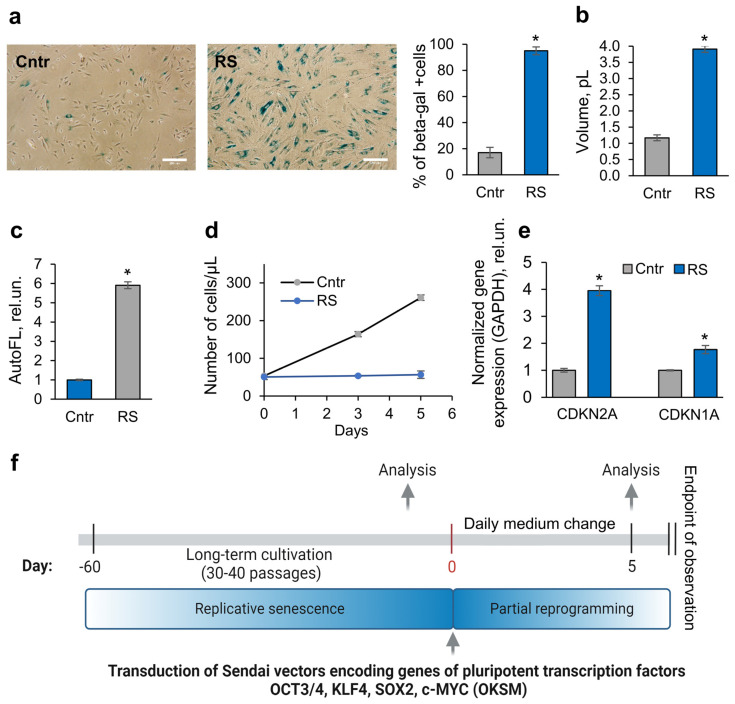
Markers of senescence in RS-MSC cultures underwent long-term cultivation. (**a**) Expression of SA-β-gal (images and quantification of the SA-β-gal+ cell fraction), scale bar = 200 µm; (**b**) increase in the mean size of the RS-MSCs; (**c**) lipofuscin-associated increase in the autofluorescence signal; (**d**) proliferation arrest evidenced by the RS-MSC growth curve; (**e**) upregulation of the marker genes of cell senescence: *CDKN2A* (codes p16) and *CDKN1A* (codes p21); and (**f**) scheme of partial reprogramming experiment. Data in B to E are shown as mean ± SD (N = 3). * *p* < 0.05 vs. Cntr. Abbreviations: Cntr, control cells (MSCs at the passage 9–11); RS, cells that underwent replicative senescence (MSCs at the passage 35–40); and MSCs, mesenchymal stem/stromal cells.

For the experiments, we chose endometrial mesenchymal stem/stromal cells as an example of MSCs because they possess all the markers and plasticity of MSCs and, in addition, can be obtained non-invasively from the menstrual blood of donors [14], which is an important factor for their potential use in therapy. Replicative senescence was achieved by the long-term cultivation of MSCs for 35–40 passages. The MSCs after prolonged cultivation (RS-MSCs) demonstrated all the features of the senescence phenotype: they expressed senescence-associated β-galactosidase (SA-β-galactosidase) (Figure 1a), enlarged their size and became flattened (Figure 1a,b), and showed enhanced autofluorescence (usually associated with the accumulation of lipofuscin in senescent cells [15]) (Figure 1c). The RS-MSC growth curve demonstrated cell proliferation arrest (Figure 1d). Moreover, we revealed the upregulation of genes, which code p21 and p16 cell cycle inhibitors (Figure 1e).

In order to check whether it is possible to rejuvenate RS-MSCs by partial reprogramming, we transduced cells with Sendai vectors containing genes encoding the classical Yamanaka transcription factors—Oct3/4, Sox2, Klf4, and c-Myc (CytoTune-iPS 2.0 Sendai Reprogramming Kit). Despite the fact that the cassette used in this study is not inducible (in contrast to those employed in most experiments to study the phenomenon of partial reprogramming), its advantage is the high efficiency and the absence of target gene incorporation into the cellular genome, thus preserving its stability. As a mock control, we used RS-MSCs that received the same handling procedures but without viral vectors. The characteristics of the partially reprogrammed RS-MSCs (PR-MSCs) and RS-MSCs were compared on the 5th day after transduction with Sendai viruses (Figure 1f), and throughout this time, the cells were cultured in standard DMEM/F12 full growth medium, which was changed daily.

#### 2.1.1. Preservation of Phenotypic Identity of PR-MSCs

**Figure 2:**
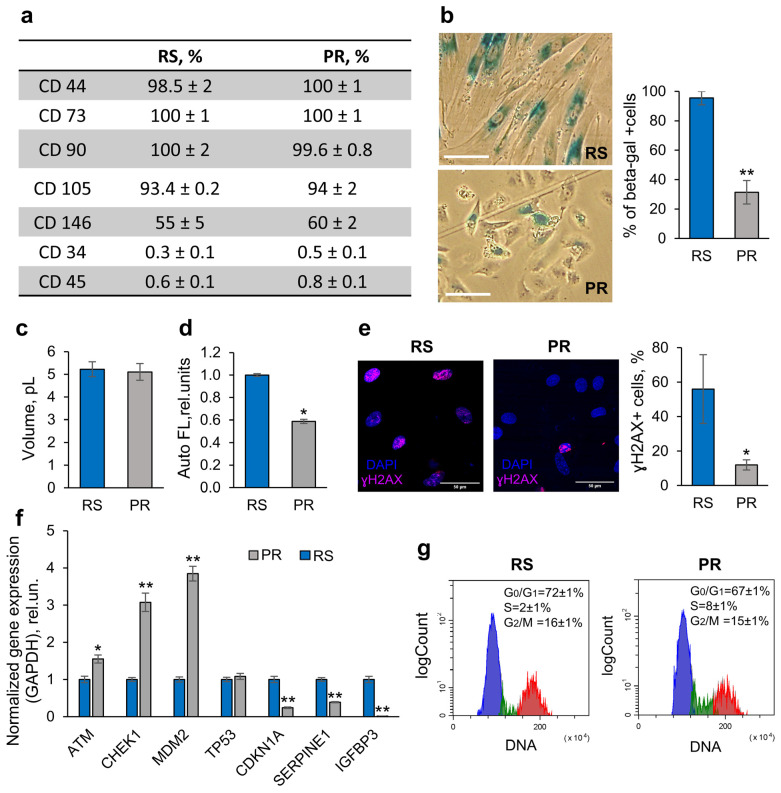
Partial reprogramming exerts a rejuvenating effect on RS-MSCs. (**a**) Immunophenotyping of RS- and PR-MSCs on MSC-specific CD markers. (**b**–**g**) Signs of senescence reversal in PR-MSCs: drop in the SA-β-gal expression, scale bar = 100 µM (**b**); measurement of the mean volume using Scepter™ 2.0 (**c**); decrease in the autofluorescence signal measured by flow cytometry (**d**); decrease in the number of cells with DNA double-strand breaks, scale bar = 50 µM (**e**); upregulation of genes involved in the DNA repair–*ATM* and *CHEK1* genes, alterations in gene expression indicating the suppressed activity of p53/p21 signaling pathway–*MDM2*, *TP53* and *CDKN1A* genes, and downregulation of genes involved in senescence propagation–*SERPINE1* and *IGFBP3* (**f**); and increase in the % of S-phase cells (G_0_/G_1_-phase (blue), S-phase (green). G_2_/M-phase (red)) (**g**). Data in (**b**–**d**,**f**,**g**) are shown as mean ± SD (N > 3). * *p* < 0.05 and ** *p* < 0.01 vs. RS. Abbreviations: RS, cells that underwent replicative senescence (MSCs at the passage 30–40); PR, partially reprogrammed cells (MSCs at the passage 30–40 after 5 days since transduction with Yamanaka factors); and MSCs, mesenchymal stem/stromal cells.

To begin with, we tested whether PR-MSCs retain their phenotypic identity during partial reprogramming. The surface CD markers were analyzed using flow cytometry. The reprogrammed cells were positive for CD90, CD105, CD73, CD 44, CD 146, and CD140 and did not express CD 34 and CD 45 (Figure 2a). Thus, we confirmed that after short-term expression of OSKM factors, the cells preserved all the phenotypic characteristic markers of MSCs.

#### 2.1.2. Change in Morphology of PR-MSCs

A change in the morphology of PR-MSCs was observed as early as day 2 after transduction with Yamanaka factors. The cell spreading decreased significantly, and the number of cells that resembled young eMSCs steadily increased and reached 85% by day 5 (visual assessment, Figure 2b). No changes in the cell size were observed when the volume of RS-MSCs and PR-MSCs was measured using the Scepter™ 2.0 Handheld Automated Cell Counter (Figure 2c); but, at the same time, partial reprogramming resulted in a significant lowering of cell autofluorescence (Figure 2d).

#### 2.1.3. SA-β-Galactosidase Activity

The number of SA-β-gal-positive cells, estimated by the quantitative processing of brightfield images using ImageJ software, markedly decreased on day 5 of the reprogramming process and constituted 31% of the cell population in comparison to 95% in the RS-MSCs (Figure 2b).

#### 2.1.4. DNA Damage

Disruption of DNA integrity is an inherent feature of senescent cells, so the next parameter evaluated was the number of DNA double-strand breaks. The immunofluorescence analysis of the γH2AX foci, a marker of DNA double-strand breaks, showed a substantial reduction in γH2AX-positive cells during partial reprogramming—from 55% observed in senescent cells to 12% in PR-MSCs on day 5 of reprogramming (Figure 2e). In addition, we found an increase in the expression level of genes, which code the proteins responsible for DNA damage repair (ATM and Chk1), that can be considered as a consequence of the DNA repair system activation (Figure 2f).

#### 2.1.5. Propagation of Senescence

Recent research [16,17] has revealed a number of highly abundant proteins in the senescence-associated secretory phenotype of endometrial MSCs used in this study. Among them, PAI-1 and IGFBP-3 have shown themselves as significant regulators of paracrine senescence progression within the endometrial MSC population [16,17]. Using RT-qPCR analysis, we found a downregulation in the *SERPINE1* (codes PAI-1) and *IGFBP3* genes that additionally testifies to the reversal of senescence-associated characteristics of PR-MSCs.

#### 2.1.6. Proliferative Activity

The arrest of cell proliferation is a key feature of cellular senescence. Using flow cytometry, we analyzed the cell cycle phase distribution of senescent and reprogrammed cells. While cycling cells were almost absent in the RS-MSCs, in the PR-MSCs the number of S-phase cells increased by day 5 (Figure 2g). In addition, the qPCR analysis revealed the upregulation of the *MDM2* gene (negatively regulates the activity of p53). In parallel, we found a significant decrease in the expression of the cyclin-dependent kinase inhibitor gene coding p21 protein, a downstream target of p53 and a marker of cellular senescence that mediates the arrest of cell proliferation (Figure 2f). The obtained results indicate that the reprogrammed cells began to restore their proliferative activity.

Based on all the abovementioned data, we can conclude that partial reprogramming erases the markers of cellular senescence and can exert a rejuvenating effect on human RS-MSCs.

### 2.2. Revitalization Is Accompanied by the Enhancement of Therapeutic Activity of PR-MSCs

**Figure 3:**
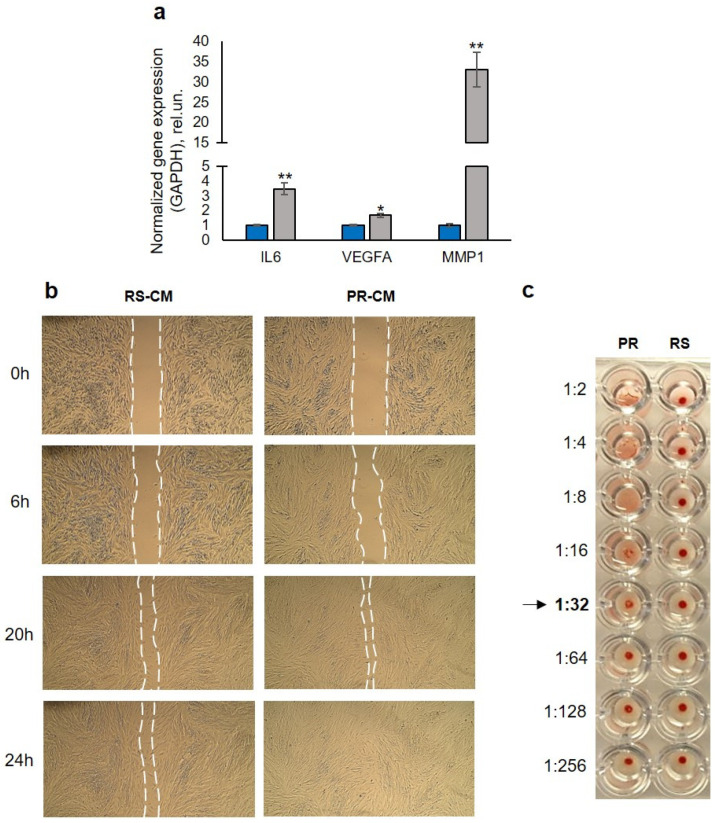
Revitalization is accompanied by the enhancement of the therapeutic potential of PR-MSCs. (**a**) RT-qPCR analysis of migration-associated genes upregulated in PR-MSCs. Data are shown as mean ± SD (N > 3). * *p* < 0.05 and ** *p* < 0.01 vs. RS; (**b**) scratch assay on MSCs using conditioned medium from RS-MSCs (left panel) and from PR-MSCs (right panel), white dotted line shows the edges of the healing scratch, scale bar = 100 µM. The most representative dynamics of scratch healing are shown. (**c**) Hemagglutination assay, testing the conditioned medium from RS- and PR-MSCs on the presence of viral components. The arrow shows the highest dilution of conditioned medium exhibiting hemagglutination. Abbreviations: RS, cells that underwent replicative senescence (MSCs at the passage 30–40); PR, partially reprogrammed cells (MSCs at the passage 30–40 after 5 days since transduction with Yamanaka factors); CM, conditioned medium; and MSCs, mesenchymal stem/stromal cells.

One of the key therapeutic features of MSCs is their ability for migration to the sites of injury to stimulate the regeneration of tissue defects, such as skin wounds. We then checked if there was an upregulation in expression of genes that stimulate cell migration of PR-MSCs. We revealed an increase in expression of *IL6* and *VEGFA*, as well as of the gene coding major matrix metalloproteinase-1 (MMP-1) (Figure 3a). By interacting with the corresponding receptors on the surface of MSCs, the first two activate signaling pathways that stimulate cytoskeleton reorganization, while MMP-1 provides remodeling of the extracellular matrix, as a whole facilitating migration and potentially contributing to wound healing and tissue regeneration processes.

In the next step, we decided to check whether revitalization is accompanied by the enhancement of the therapeutic paracrine potential of PR-MSCs. For this purpose, we collected the conditioned medium produced by both senescent and revitalized cells over a 24 h period, from day 4 to day 5 of reprogramming. Exploiting this medium, we performed an in vitro wound-healing assay using the same endometrial MSC line as for the partial reprogramming experiments but on the early passage. The obtained data demonstrated accelerated scratch overgrowth in the case of the medium from the PR-MSCs compared to the medium from the RS-MSCs (Figure 3b). This fact suggests that even short-term activation of pluripotent transcription factors may result in the enhancement of MSC therapeutic activity, which is impaired after prolonged in vitro expansion of the cell culture. Having come to this conclusion, we further tested the conditioned medium from revitalized MSCs for biosafety. Sendai virus, which was used for RS-MSC transduction, is a murine parainfluenza virus, expressing a hemagglutinin protein on the surface of its envelope, which can cause agglutination of erythrocytes. By carrying out a hemagglutination assay using chicken blood as a source of erythrocytes, we assayed the conditioned medium for the presence of the viral components. The conditioned medium from PR-MSCs demonstrated hemagglutination activity (with a titer equal to 32 hemagglutination units), which resulted in the formation of a lattice (diffuse reddish solution) even in highly diluted samples, in contrast to samples from RS-MSCs, in which erythrocytes precipitated (dark red pellet) (Figure 3c). Collectively, our data suggest that partial reprogramming is a potentially effective strategy for the rejuvenation of cultured MSCs in late passages but requires an alternative strategy of reprogramming (for example, chemical reprogramming [18]) for biomedical applications of rejuvenated cells.

## 3. Discussion

The partial reprogramming technique has shown promising results in the amelioration of the senescence-associated cell phenotype in vitro and in vivo [11]. The studies carried out were mostly aimed at the rejuvenation of experimental animals or cells isolated from aged donors (mice and humans). However, the development of senescence in cell cultures is also a serious issue concerning biomedical applications of human cells, especially MSCs. At the same time, to the authors knowledge, there are very few data regarding the partial reprogramming of cultured human MSCs. In the present study, we attempted to use this method to rejuvenate human MSCs that had undergone replicative senescence in culture. We aimed to test whether it is possible to consider cellular revitalization as a strategy for obtaining a large biomass of therapeutically active human cells after their long-term propagation. The reversal of cellular senescence in culture would make it possible to obtain an unlimited source of isogenic cells suitable for use in regenerative medicine, including the fabrication of cell products/tissue-engineered constructs and accumulation of therapeutically active conditioned medium.

At the first stage, we subjected human MSCs that underwent replicative senescence to partial reprogramming. The experiments proved the possibility of rejuvenation of MSCs that had completely lost their proliferative activity after 40–50 cycles of cell population doubling in culture. Reprogramming was performed by the transduction of classical Yamanaka transcription factors—Oct3/4, Klf4, Sox2, and c-Myc (OKSM). Nowadays, many methods for the delivery of pluripotency factors have been developed. The most studied and widespread is the delivery of pluripotency factors using retroviruses; however, this method carries risks, such as insertional mutagenesis, residual expression, and reactivation of reprogramming factors [19]. Alternative methods for delivering reprogramming factors are RNA transfection, transient or episomal transfection [13,20], and chemical-based reprogramming using small molecules and growth factors [18], as well as lipophilic compounds [21]. In this work, we employed Sendai virus for OKSM delivery because this vector possesses the highest reprogramming efficiency among non-integrative protocols, and all the processes associated with its replication/protein synthesis occur in the cell cytoplasm without affecting the nucleus [22]. Typically, the duration of one cycle of partial reprogramming to achieve the rejuvenation effect in different studies varies from 2 days to 2.5 weeks, which is largely due to a balance between attempts to enhance the revitalization effect and prevent the loss of the original cellular phenotype [11]. Our results showed that the expression of the OKSM factors in senescent human MSCs for five days is sufficient for a dramatic decrease in the markers of cellular senescence while maintaining the somatic identity of the cells. Despite all the advantages of the Sendai-based reprogramming system used in this study, it does not allow for interrupting the reprogramming process; so, within this experimental design, we were unable to further follow the fate of revitalized cells. Nevertheless, the data obtained in our work allow one to use the partial reprogramming technique to study the effect of cellular revitalization and consider PR-MSCs as a suitable model in fundamental research of aging. It is worth noting that, at first glance, our data seem to contradict the findings of Göbel et al., who also used short-term expression of Yamanaka factors to address MSC senescence [13]. However, we would like to point out the differences in our approaches and reprogramming methods. The study by Dr. Göbel and colleagues was aimed at finding the way to delay the onset of senescence in MSCs, whereas ours focused on reversing already developed MSC senescence; thus, we cannot directly compare the obtained results.

After proving the erasure of the senescence-associated characteristics of the PR-MSCs, we assessed whether revitalization can lead to the enhancement of the therapeutic potential of these cells. We showed that the partial reprogramming protocol employed in this study resulted in the increase in gene expression of a number of factors stimulating cell migration—an important aspect of MSC functioning within a human body. Along with MSCs themselves, conditioned MSC medium without a cellular component appears to be another promising tool for use in regenerative medicine. Consequently, the next stage of our work was focused on evaluating whether partial reprogramming can restore the therapeutic activity of the conditioned medium from senescent MSCs. Having carried out an in vitro scratch assay, we found that the medium from PR-MSCs, in comparison with the medium from RS-MSCs collected after 24 h incubation from the 4th to the 5th day of reprogramming, causes accelerated healing of the scratch on the cell monolayer. This can be evidence of the improvement in the wound-healing potential of the medium conditioned by revitalized cells and allows us to consider these mediums as therapeutically active substances. However, in the next set of experiments, we tested the biosafety of the media from PR-MSCs using a hemagglutination assay. The test detected the formation of erythrocyte clumps in samples from PR-MSCs, indicating the presence of hemagglutinin in this medium. Since the cell medium was changed daily during the 5-day partial reprogramming, we assume that a hemmagglutination reaction is more likely due to hemagglutinin, either free or bound to membrane residues, rather than to remaining viral particles. The results obtained prove that the use of viral infection as a method of MSC revitalization may hinder the therapeutic use of media conditioned by revitalized cells. Because exploiting viral infection for rejuvenation may carry a risk for the application of PR-MSCs in clinical practice, alternative methods of cellular reprogramming are needed for biomedical applications using revitalized cells. An approach with great potential may be a chemical reprogramming technique [18].

To conclude, in the present study, we have proven the possibility of the rejuvenation of human MSCs that underwent replicative senescence in culture using partial reprogramming, created a biological model of replicative senescence/revitalization, and showed that the approach used can provide the potential benefits for the improvement in the therapeutic activity of the conditioned MSC medium. At the same time, our experiments have proven the biological danger of medium conditioned by rejuvenated MSCs when using a viral infection for the delivery of reprogramming factors. Testing various combinations of reprogramming factors, including chemical ones; altering the duration and cyclicity of the reprogramming process; and studying the rate of senescence phenotype reacquiring after the reprogramming was stopped—all these studies are necessary to create an optimized and safe protocol that will allow for the future use of the partial reprogramming strategy in cell therapy.

## 4. Materials and Methods

### 4.1. Cell Cultures

Mesenchymal stem/stromal cells (MSCs). Human MSCs have been previously derived in our laboratory from human desquamated endometrium (view in [14]). The MSCs were cultivated in DMEM/F12 medium (Gibco, Waltham, MA, USA) supplemented with 10% FBS (HyClone, Washington, DC, USA), 1% L-glutamine, and 1% penicillin-streptomycin (Gibco, Waltham, MA, USA). The cells were maintained at 37 °C, 5% CO_2_, and were routinely subcultured twice a week at a split ratio of 1:3.

### 4.2. Induction of Cell Senescence and Rejuvenation

MSC senescence. To achieve replicative senescence, the MSCs were cultivated in standard growth conditions up to the 35–40th passage (50–60 cycles of cell population doubling). The cultivated cells were reseeded after reaching a subconfluent density. Up to the 25th passage, the MSCs were typically subcultured twice a week at the split ratio of 1:3 and later, at the split ratio of 1:2, at first twice and then once a week. The MSCs that achieved replicative senescence stopped their proliferation.

MSC rejuvenation. The cells were rejuvenated after long-term cultivation (at the 35–40th passage) using the partial reprogramming technique. Partial reprogramming was carried out by the ectopic expression of the Yamanaka factors (Oct3/4, Sox2, Klf4, and c-Myc) in senescent MSCs with the use of the CytoTune-iPS 2.0 Sendai Reprogramming Kit (Thermofisher Scientific, Waltham, MA, USA). The kit includes three Sendai vectors, polycistronic Klf4–Oct3/4–Sox2 (KOS), cMyc, and Klf4, and provides non-inducible permanent production of Yamanaka factors in cell cytoplasm. For the experiments, senescent MSCs were seeded in the 3 cm dishes the day before transduction (100,000 cells per dish) in DMEM/F12 medium (Gibco, Waltham, MA, USA) supplemented with 10% FBS (HyClone, Washington, DC, USA), 1% L-glutamine, and 1% penicillin-streptomycin (Gibco, Waltham, MA, USA). On day 0, the cell medium was changed to a fresh DMEM/F12 (Gibco, Waltham, MA, USA) containing three Sendai viruses. The amount of viruses added was calculated based on the virus titers listed in the manufacturer’s instruction at MOI = 5:5:3 (KOS:c-Myc:Klf4). On day 1, the cell medium containing the residual viruses was replaced by a fresh full growth DMEM/F12 medium. From day 1 to 5, the full growth DMEM/F12 medium was changed daily. All the assays on the reprogramming cells were performed on the 5th day after transduction, enabling the characterization of cells after only short-term expression of the Yamanaka factors.

### 4.3. Senescence-Associated Β-Galactosidase Staining

The senescent MSC cultures were assayed for the expression of the senescence-associated β-galactosidase (SA-β-gal) with the use of the Senescence β-Galactosidase Staining Kit (Cell Signaling Technology, Boston, MA, USA) according to the manufacturer’s instructions. For each sample, not less than 200 randomly selected cells were analyzed.

### 4.4. Autofluorescence

The increase in the cell autofluorescence (EX488/FL525 signal), which is usually associated in the senescent cells with the lipofuscin accumulation, was detected with the use of a CytoFLEX flow cytometer (Beckman Coulter, Brea, CA, USA).

### 4.5. Cell Proliferation

The arrest of cell proliferation in senescent MSCs was confirmed by measuring the growth curves with the use of the CytoFLEX flow cytometer (Beckman Coulter, Brea, CA, USA). In these experiments, only viable cells identified by the propidium iodide staining were counted. For the cell cycle analysis, the cells were harvested and permeabilized with 0.1% Triton X-100 (Sigma-Aldrich, St. Louis, MI, USA) and stained for 5 min with 2 μg/mL of 4,6-diamidino-2-phenylindole (DAPI, Sigma-Aldrich, St. Louis, MI, USA) at room temperature. The cell cycle phase distribution was assessed using flow cytometry (CytoFLEX, Beckman Coulter, Brea, CA, USA, 405 nm laser) with the subsequent analysis by CytExpert 2.0 (Beckman Coulter, Brea, CA, USA) software.

### 4.6. Cell Size Measurement

The cell volume was estimated in pL with the use of the Scepter™ 2.0 Cell Counter (Merck Millipore, Burlington, Burlington, NJ, USA).

### 4.7. Immunophenotyping Analysis

The MSCs were harvested using 0.05% trypsin/EDTA solution. The cells in an amount of 10^6^/mL were suspended in PBS with 5% fetal bovine serum and incubated with antibodies to CD90, CD105, CD73, CD 44, CD 146, CD140, CD 34, and CD 45 conjugated with phycoerythrin (PE) at 4 °C for 1 h, and then the surface marker expression was measured by flow cytometry (CytoFLEX, Beckman Coulter, Brea, CA, USA).

### 4.8. RT-qPCR Analysis

The RT-qPCR analysis was performed as described in [23]. The list of primers used is given in Table 1. Expression of the target genes was normalized to the expression of the GAPDH housekeeping gene. All the amplification reactions were performed in triplicates.

### 4.9. Immunofluorescence Analysis

An immunofluorescence analysis was performed as described in [23]. Primary antibodies: anti-ɣH2AX (1:500, Abcam, Cambridge, UK); secondary antibodies: GAM Alexa Fluor 488 (Thermofisher Scientific, Waltham, MA, USA). The coverslips were imaged using confocal laser scanning microscopy (Olympus FV3000, Olympus Corporation, Tokyo, Japan).

### 4.10. Wound-Healing Assay

The conditioned medium containing 10% fetal bovine serum was collected after 24 h incubation from the 4th to the 5th day after induction of the reprogramming from the transduced and control senescent MSCs. The collected medium was then centrifuged on 3000× *g* to remove the cell debris and stored at −80 °C until use. Two-well silicone inserts (Ibidi, Graefelfing, Germany) in 24-well plates were used to create a defined cell-free scratch. A total of 2 × 10^4^ MSCs were seeded on both sides of the insert and incubated overnight to reach a confluent monolayer. Then, the inserts were removed, the cells were washed with PBS, and 800 µL of conditioned medium from partially reprogrammed or control senescent cells was added to the well. Phase-contrast MSC images were taken at 0, 6, 20, and 24 h to monitor the gap closure.

### 4.11. Hemagglutination Assay

Firstly, a serial 2-fold dilution of conditioned medium used for the wound-healing assay was performed with 0.9% saline solution in a U-bottom-shaped 96-well immunological plate (Nunc, Waltham, MA, USA), starting with 100 µL of undiluted conditioned medium in the first row. After that, 50 µL of 1% chicken blood suspension in 0.9% saline solution was added to each well and mixed. Then, the plate was incubated for 30 min at 4 °C. The appearance of a dark red pellet is evidence of the absence of agglutination in a sample, while the formation of a lattice indicates the presence of the erythrocyte/viral hemagglutinin clumps. The hemagglutination titer is reciprocal of the highest dilution of conditioned medium exhibiting hemagglutination.

### 4.12. Statistics

All the experiments were repeated at least 3 times. The data are presented as the means ± SD, when indicated. The statistical significance in the pairwise comparisons was evaluated by Student’s t-test, and *p* < 0.05 was considered to be significant. The microscopy images and flow cytometry histograms shown correspond to the most representative experiments.

## Figures and Tables

**Table 1:** The list of primers used for the qPCR assay.

Gene	Primer Sequence	Annealing Temperature (T °C)
*CDKN1A*	F: 5′-CCACATGGTCTTCCTCTGCTG-3′ R: 5′-GATGTCCGTCAGAACCCATG-3′	61
*CDKN2A*	F: 5′-GAGCAGCATGGAGCCTTC-3′ R: 5′-CCTCCGACCGTAACTATTCG-3′	58
*MDM2*	F: 5′-TGGGCAGCTTGAAGCAGTTG-3′ R: 5′-CAGGCTGCCATGTGACCTAAGA-3′	62
*CHEK1*	F: 5′-ACCCCAGGATCCTCACAGAA-3′ R: 5′-AGCAGCACTATATTCACCAGGA-3′	62
*ATM*	F: 5′-CAGGCGAAAAGAATCTGGGG-3′ R: 5′-GCACAAAGTAGGGTGGGAAAGC-3′	62
*GAPDH*	F: 5′-GAGGTCAATGAAGGGGTCAT-3′ R: 5′-AGTCAACGGATTTGGTCGTA-3′	59–62
*TP53*	F: 5′-CCTCAGCATCTTATCCGAGTGG-3′ R: 5′-TGGATGGTGGTACAGTCAGAGC-3′	60
*IGFBP3*	F: 5′-TCACCTGAAGTTCCTCAATGT-3ʹ R: 5′- ACTTATCCACACACCAGCAGA-3ʹ	60
*SERPINE1*	F: 5′-CTCATCAGCCACTGGAAAGGCA-3′ R: 5′-GACTCGTGAAGTCAGCCTGAAAC-3′	60
*IL6*	F: 5′-AAGCCAGAGCTGTGCAGATG-3′ R: 5′-GTCCTGCAGCCACTGGTTCT-3′	60
*VEGFA*	F: 5′-CTACCTCCACCATGCCAAGT-3′ R: 5′- GATAGACATCCATGAACTTCACCA-3′	60
*MMP1*	F: 5′-ACAGCTTCCCAGCGACTCTA-3′ R: 5′-TTGCCTCCCATCATTCTTCAGG-3′	60

## Data Availability

The data will be made available on request.
